# Early Exposure and Its Impact on Cardiothoracic Surgery: an Experience of Medical Education in The United Kingdom

**DOI:** 10.21470/1678-9741-2020-0487

**Published:** 2022

**Authors:** Jeremy Chan, Ka Siu Fan, Hiu Tat Kwok, Shwe Oo

**Affiliations:** 1 Faculty of Health Sciences, Bristol Medical School, University of Bristol, Bristol, United Kingdom.; 2 Faculty of Medicine, St. George’s Medical School, University of London, London, United Kingdom.; 3 Department of Geriatrics, Queen’s Medical Centre, Nottingham, United Kingdom.; 4 Bristol Heart Institute, Bristol, United Kingdom.

**Keywords:** Cardiothoracic Surgery, Education, Medical, Undergraduate, Training, Curriculum

## Abstract

**Introduction:**

Cardiothoracic surgery (CTS) has seen a decline in interest and application rates in recent years. As a relatively small speciality, teaching and placements in CTS are often not included during undergraduate study and postgraduate training. We aim to evaluate the exposure to CTS during both undergraduate study and postgraduate training.

**Methods:**

A ten-question online survey was designed and delivered to Foundation Year Two (FY2) doctors who graduated in 2017 and completed their two-year postgraduate foundation training in 2019. Medical schools with no graduates in 2017 and 2018 were excluded from our study. IBM® SPSS Statistics, version 25, and Microsoft Excel 365® were used for Student’s *t*-test statistical analysis.

**Results:**

Three hundred and six FY2 doctors across 16 medical schools completed the survey, none of which included compulsory CTS attachments as their undergraduate curriculum. Thirty-two respondents (10.5%) underwent CTS attachments lasting between one to three weeks. Only 14 (43.8%) had worked in a cardiothoracic unit during their two-year Foundation Programme; 10 of which (71.2%) subsequently made an application for cardiothoracic speciality training. Most of the participants with previous exposure to CTS, during either undergraduate study or postgraduate Foundation Programme training or both, were significantly more likely to make an application to CTS training (*P*<0.05).

**Conclusion:**

Our study suggests that doctors with increased exposure to CTS during undergraduate study and postgraduate training are more likely to pursue a career in CTS. Targeted interventions at both stages may improve interests in CTS and the number of prospective applicants.

**Table:** Abbreviations, acronyms & symbols

CTS	= Cardiothoracic surgery
FY2	= Foundation Year Two
PBL	= Problem-based learning
ST1	= Speciality Training Year 1
ST3	= Speciality Training Year 3
UK	= United Kingdom
VATS	= Video-assisted thoracoscopic surgery

## INTRODUCTION

Interests and application rates for cardiothoracic surgery (CTS) have fallen in recent years. The overall volume of applications for both early (speciality training year 1 [ST1]) and intermediate (speciality training year 3 [ST3]) cardiothoracic training has undoubtedly declined^[1,2]^. As a relatively small speciality, CTS is often not included in undergraduate study and postgraduate training which may be contributing to its decreasing interests. A study by Woolf et al.^[3]^ has suggested that both undergraduate and postgraduate exposures are essential factors in evaluating long-term career choice for junior doctors. Similarly, Goldacre et al.^[4]^ identified that eventual career choices of junior doctors were heavily influenced by their experience as a student and their experience with the particular speciality departments during medical school. As suggested by the study by Shaikh et al.^[5]^, additional teaching in the undergraduate curriculum to medical students increased understanding of the speciality and helped foster student interests in pursuing a career in that speciality.

Within the United Kingdom (UK), exposure to medical and surgical specialities typically begins during the clinical phase of medical school where they have two to three years to develop career directions. Upon graduating, they become junior doctors and work under two years of Foundation Programme where they receive further exposure to specialities of interest before committing to specific training pathways. This means junior doctors would typically have less than four years to decide and commit to a speciality by the end of Foundation Year 2 (FY2). This results in the indecisiveness in career paths of many medical students and junior doctors. A 2011 London-based survey found that only 10% of final-year students had a definitive career choice, with the majority yet to commit to a speciality and up to 15% being ‘undecided’^[6]^. This concurs with a similar survey in the early 2000s which also found 15% of final year students undecided on career paths^[7]^. It is also important to note that students who have interests in particular specialities often continue to pursue training in the same field as a junior doctor^[8]^.

These findings show career uncertainty as a longstanding issue to be addressed as they will inevitably be carried into their careers during and after the Foundation Programme. With the 2018 Foundation Programme report detailing 18.8% of Foundation Year 1 doctors as undecided on career choice, the situation will likely be exacerbated by the limited exposure to various specialities during the Foundation Programme^[9]^. Like many surgical specialities, doctors that show early and substantial commitment are more likely to excel in CTS, hence, early exposure is ever more important. Therefore, this study was conducted to evaluate exposure to CTS both during undergraduate study and postgraduate training.

## METHODS

A ten-question survey was designed and delivered to FY2 doctors who graduated in 2017 and completed their two-year postgraduate foundation training in 2019. The details of the survey are shown in Table 1. The survey focused on: a) student exposure to CTS within their undergraduate curriculum, b) student exposure to CTS outside their undergraduate curriculum, c) exposure to CTS as a doctor within their postgraduate foundation training, d) foundation doctor exposure to CTS outside of postgraduate foundation training, and e) their self-reported interests in CTS.

**Table 1:** Ten-question survey.

1. Medical schools and year of graduation:
2. Have you undertaken a placement in cardiothoracic surgery during your undergraduate study?
3. If yes, how long was the placement?
4. Have you undertaken any additional placement during your undergraduate study? (*e.g*., student selected components or electives)
5. Is there any additional/private teaching in cardiothoracic surgery during your undergraduate study?
6. If yes, what was the method of teaching? (please circle)
7. Lectures/Bedside teaching/Small group tutorials
8. Have you worked, as part of your formal rotation, in cardiothoracic surgery during your postgraduate foundation training?
9. Have you worked, NOT as part of your formal rotation, in cardiothoracic surgery during your postgraduate foundation training? (*e.g*., taster weeks)
10. If yes, have you performed (under supervision) the following procedures? (please circle)
11. Saphenous vein harvesting/Sternotomy Open + Closure/Thoracotomy Open + Closure/ Any VATS procedures
12. Did you make an application to cardiothoracic surgery speciality training after completion of foundation year training?

VATS=video-assisted thoracoscopic surgery

The survey was delivered online to junior doctors in 2019. The study was conducted using the SurveyMonkey® platform and was distributed through junior doctor social media groups and university societies. Medical schools with no graduates in 2017 and 2018 were excluded from our study. IBM Corp. Released 2017, IBM SPSS Statistics for Windows, Version 25.0, Armonk, NY: IBM Corp. and Microsoft Excel 365® were used for data collection and Student’s t-test statistical analysis. A *P*-value < 0.05 is deemed statistically significant.

## RESULTS

**Fig. 1:**
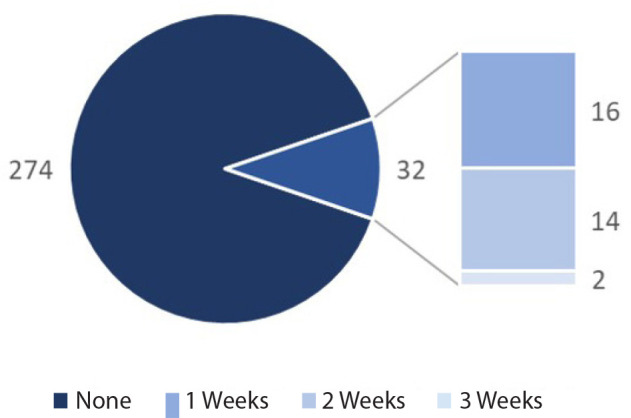
The reported length of cardiothoracic placements.

A total of 306 fully completed surveys from FY2 doctors across 16 medical schools in England, Northern Ireland, and Wales were received. No respondents reported having compulsory CTS placements. Thirty-two respondents (10.5%) underwent an attachment in CTS during medical school, ranging between one and three weeks in total. A breakdown of CTS placements is shown in Table 2 and Figure 1.

**Table 2:** Breakdown of graduate origins and cardiothoracic placements.

Nation	Medical Schools	Respondents	CTS Placements
England	13	110	14 (12.7%)
Northern Ireland	1	122	6 (4.9%)
Wales	2	74	12 (16.2%)

CTS=cardiothoracic surgery

**Fig. 2:**
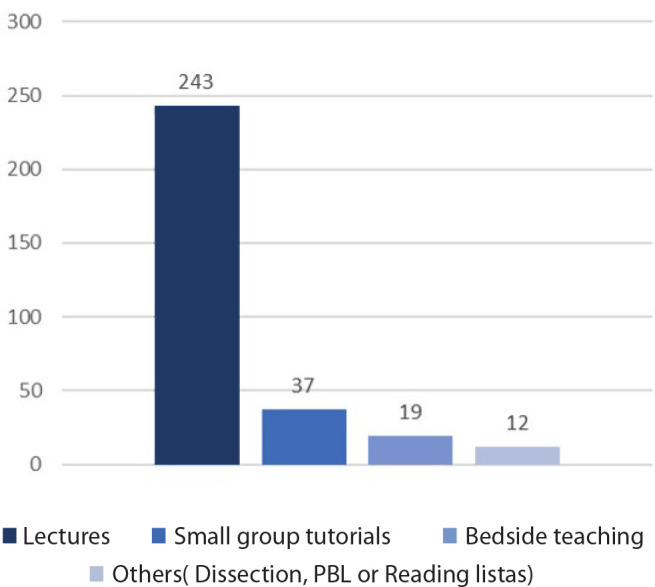
Breakdown of the teaching methods for cardiothoracic surgery. PBL=problem-based learning

Three medical schools were reported to provide a one-week placement that allowed access to CTS integrated into their cardiovascular or respiratory medicine placement blocks. One hundred and sixty-one (52.61%) had received organised teaching on CTS. This was mainly in the form of physical/online lectures, followed by small group tutorials and bedside teaching. A breakdown of commonly reported teaching methods is shown in Table 3 and Figure 2.

**Table 3:** Types of cardiothoracic surgery teaching received at undergraduate levels.

Nation	Lecture	Small group tutorials	Bedside teaching	Others*
England	73	37	11	2
Northern Ireland	122	0	0	0
Wales	48	0	8	10

* Dissection, problem-based learning, or reading lists

In terms of postgraduate exposure, 14 individuals subsequently worked in a cardiothoracic unit during their two-year Foundation Programme training. Of this group, ten (71.2%) respondents later made an application for cardiothoracic speciality training. Of those that had CTS attachments during undergraduate training, but not during the Foundation Programme, six (33%) made an application for cardiothoracic speciality training. All 14 respondents had performed saphenous vein harvesting and video-assisted thoracoscopic surgery. Only a limited number of FY2 doctors (n=3) had performed a sternotomy or thoracotomy.

Junior doctors with previous exposure to CTS, during either undergraduate study or postgraduate foundation training, or both, were more likely to make an application to cardiothoracic speciality training (*P*<0.05).

## DISCUSSION

CTS has seen a decline in interest and application rates in recent years; ST3 applications peaked at an excess of 150 per year in the late 2000s but fell steadily to below 40 in 2018^[2]^. While this is partially due to the implementation of a ‘run-through’ programme for entry via ST1, the overall volume of applications for cardiothoracic training had undoubtedly declined even when accounting for ST1 applications. This phenomenon may be reflective of the knowledge and exposure to CTS, as well as changes in attitude towards the speciality in recent years.

Our study indicated that both postgraduate and undergraduate CTS exposures are limited in the UK. Responses indicate that none of the 16 included medical schools had compulsory or dedicated CTS clinical placement. Hence, only 32 respondents have had CTS attachments during their undergraduate studies, with most lasting one to two weeks. Additionally, only 14 respondents had worked in a cardiothoracic unit during Foundation Programme training. Despite a limited number of applicants, junior doctors with exposure to CTS are more likely to make an application for CTS training (*P*<0.05). The findings of this study may be able to guide further interventions to stimulate engagement in the speciality.

### Factors Affecting Interests in CTS and Career Choice

Our current knowledge of CTS interests and attitudes of medical students can be summarised by two recent national surveys. They examined medical students’ attitude towards CTS and found that approximately 27-31% of the cohort was considering a career in surgery^[10,11]^. However, the numbers may be as low as 14% among final-year students. The surveys also highlight the disparity between CTS experiences both between and within individual medical schools. The survey by Preece et al.^[10]^ revealed that 75% of students had inadequate to no exposure to CTS within the curriculum and only 13% have attended a cardiothoracic conference or careers day. Contrarily, the survey by Gasparini et al.^[11]^ found that 71% of the students surveyed have had CTS exposure within the curriculum, with many receiving CTS opportunities through surgery (45.1%), lectures (45.1%), and conferences (42.6%).

Additionally, other motivators and barriers may affect CTS engagement of students and junior doctors. A survey by Sutton et al.^[12]^ examined 482 students from 20 medical schools and identified the two most important factors as interests in the speciality and its work-life balance. The increased exposure during clinical attachments and dedicated careers lectures can facilitate these factors and can be beneficial to careers choices at the undergraduate level. These findings are also identified in other specialities and countries as suggested by the survey and review of plastic surgery application in Canada by Austin et al.^[13]^ They identified that more than 44% of students, who were not considering plastic surgery as a career, indicating that increased exposure to the speciality will increase their interests.

### Mentorship

With similar trends in CTS observed in the United States of America, Allen et al.^[14]^ have studied the impact of mentorship and increased involvement of medical students in both laboratory research and surgery. Through increasing shadowing opportunities, involvement in animal-based operations, and clinical research projects, the majority of students applied for surgical subspecialities, with as many as 20% towards CTS. They identified that the presence of mentors greatly influences career decisions by providing insight into CTS — and surgery as a whole —, ultimately recommending the establishment of programmes to facilitate the engagement of medical students during medical school. This is also supported by the work of Woolf et al.^[3]^ in establishing the effects of undergraduate and postgraduate exposures on subsequent career choice. Implementation of outreach or mentorship programmes such as the academic mentoring programme trialled by Fricke et al.^[15]^ can also be beneficial as supported by the survey where respondents feel the best way to promote CTS is to directly observe CTS (46%) and meeting cardiothoracic surgeons (15%)^[11]^. Similarly, to address the lack of knowledge regarding the nature and pathway of CTS training, basic introduction to the fundamentals for all specialities should be provided at medical school to help foster their interests and facilitate informed career decisions^[11]^.

### Types of Exposure and Teaching

These findings highlight that interventions in both undergraduate curriculum and extracurricular activities are required to rectify the declining interest in CTS. In a recent systematic review of factors affecting choosing surgery as a career, 11 of the 21 studies indicated that clinical exposure improves both knowledge and interests in surgery^[16]^. While exposure can change the perception of surgery and its lifestyle, a study on neurosurgery reported that it reduced interests. One of the concerns identified by Zuccato et al.^[17]^ was the need to make early career commitments which leaves other specialities unexplored, leading to reconsiderations. This phenomenon is likely also applicable to CTS as it also requires dedication in the speciality early on.

Regardless of its effects on career choices, the accessibility to CTS, like other smaller specialities, is often limited to large high-volume centres. Of those that are affiliated with medical schools, they may not offer enough placement opportunities to accommodate the number of students. This results in very limited opportunities for students to gain exposure and scrub in theatres. This is supported by data from our study, in which only 10.46% of students had a placement in CTS. While our finding may appear lower than those of Gasparini et al.^[11]^, it may be attributable to more extensive inclusion criteria than using clinical placement alone in this study. As such, most UK medical schools are unable to include compulsory CTS placements for all students, driving the majority of cardiothoracic teaching into non-clinical environments. These activities include lectures and small group teachings on cardiothoracic emergencies such as aortic dissection, in which all medical schools have covered as part of the curriculum, with lectures being the primary mode of delivery. Other methods may be considered to influence interests and education, such as the use of short workshops as studied by ElHawary et al.^[18]^. Their study assessed the effect of a 60-minute suturing workshop for medical students on their confidence and interest in surgery. Of the 85 medical students that participated, 82% reported being more interested in surgery after the workshop, demonstrating that even short-lasting, practical experiences are also beneficial. Similarly, interactive sessions from one-day skills day are also effective at improving understanding of surgery and changing student perceptions of surgery^[19]^. Furthermore, both surgical simulation and three-dimensional modelling are increasingly recognised methods that may also have a place in improving student surgical interests^[20-23]^. Regardless, despite being highly valued and commonly utilised pedagogic methods, literature have emphasised the unique benefits of clinical attachments and the importance of students being part of a team^[24]^.

In short, it is difficult to foster interests in CTS through clinical exposure alone, and other alternatives should also be considered. The increasing demand for the ageing population must be met by attracting prospective cardiothoracic surgeons^[25,26]^. Our findings are supported by similar studies across other specialities which identify exposure as an important factor towards surgical interests. This study has highlighted the need to implement various interventions at both undergraduate and postgraduate levels to anticipate and address the future demands of the speciality.

### Limitations

The primary limitations of this study lie in its survey-based methodology, which provides an estimation of respondents who are interested in CTS. As such, respondents from only 16 medical schools have been captured and is not wholly representative of all medical students and foundation doctors in the UK, especially as there were no Scottish graduates who responded. The voluntary nature of surveys can also introduce response bias as doctors with interests in CTS or early exposure may be more likely to respond, producing an overestimate of CTS teaching and exposure. While conducting this survey online helps maximise respondents reached, this method prevents the calculation of response rates as it is not known how many this survey has reached. As this is a cross-sectional survey, it cannot account for the pre-existing interests in CTS which can affect whether the respondents choose to apply for CTS training. Additionally, as the curriculum of undergraduate study and postgraduate training are constantly changing to meet standards of the General Medical Council, reported experiences in this survey may not hold for later cohorts. Further studies should also consider including demographic information of participants’ gender, ethnicity, and socioeconomic status as these may also affect interests in surgical training^[16,27]^.

## CONCLUSION

Our study found that students with exposure to CTS in undergraduate study and postgraduate training are more likely to pursue a career in CTS. However, CTS remains a small speciality and its interests are limited to a small cohort of students/junior doctors. We also found variations in CTS placement opportunities both within and between medical/foundation schools which can vastly differ and leave a lasting impact on interests towards CTS. Although it may not be logistically feasible to ensure CTS exposure for all students/doctors, more can be done to promote awareness by increasing student exposure through extracurricular activities such as mentorship and research.
